# Euglycemic Diabetic Ketoacidosis Presenting With Intraoperative Cardiac Arrest in Late Pregnancy

**DOI:** 10.7759/cureus.106959

**Published:** 2026-04-13

**Authors:** Luis F Gonzalez Vazquez, Angel Diaz Sanchez, Yelka Matos Furones, Paola Vanessa Sosa Sarmiento, Jose I Gamboa Arisso, Elizabeth Blanco Espinosa

**Affiliations:** 1 Anesthesiology, Geisinger Medical Center, Danville, USA; 2 North Georgia Clinical Research/Neurology, Alcanza Clinical Research, Woodstock, USA; 3 Geriatrics, Universidad del Valle, Cali, COL; 4 General Medicine, Jimmy Hirtzel Hospital, Miami, USA; 5 Neurosurgery, Hospital Arnaldo Milian, Villa Clara, CUB; 6 General Practice, Ceda Orthopedic Group, Miami, USA; 7 General Surgery, Hospital Arnaldo Milian, Villa Clara, CUB

**Keywords:** cardiac arrest, cesarean section, euglycemic diabetic ketoacidosis, pregnancy complications, type 1 diabetes mellitus

## Abstract

Euglycemic diabetic ketoacidosis (EDKA) is an uncommon but serious metabolic complication in pregnancy, where physiologic changes promote ketone production even without marked hyperglycemia. We report the case of an 18-year-old woman at 35 weeks and 4 days of gestation with type 1 diabetes mellitus and severe preeclampsia who developed intraoperative cardiac arrest during an emergent cesarean delivery. She had been nonadherent with insulin therapy for more than seven days before admission. Initial laboratory evaluation demonstrated metabolic abnormalities despite normoglycemia. During cesarean delivery, inadequate epidural anesthesia required rapid sequence intubation. Following delivery and administration of tranexamic acid for hemorrhage control, the patient developed progressive bradycardia, hypotension, and pulselessness. Cardiopulmonary resuscitation was initiated, and return of spontaneous circulation occurred within one minute. Post-arrest laboratory studies revealed severe metabolic acidosis, markedly low bicarbonate, elevated anion gap, and ketonuria consistent with EDKA. Magnesium levels were elevated but below thresholds typically associated with severe toxicity. This case highlights how overlapping obstetric, anesthetic, and metabolic stressors can obscure the diagnosis of EDKA and precipitate acute cardiovascular instability. Early recognition of acid-base disturbances and prompt multidisciplinary management are essential in high-risk obstetric patients, particularly when insulin omission and advanced gestation increase susceptibility to ketosis.

## Introduction

Euglycemic diabetic ketoacidosis (EDKA) is an uncommon but serious metabolic complication in pregnancy, characterized by significant ketosis and metabolic acidosis despite normal or only mildly elevated blood glucose levels, typically defined as <250 mg/dL. In the general diabetic population, EDKA accounts for approximately 2-3% of all diabetic ketoacidosis (DKA) cases; however, its incidence is higher in pregnancy due to unique metabolic and hormonal adaptations that promote accelerated ketogenesis even in the absence of marked hyperglycemia [1]. Although DKA complicates only 1-3% of pregnancies affected by diabetes, it remains one of the most severe acute metabolic emergencies in obstetric medicine and is associated with substantial maternal and fetal morbidity [2]. Historically, fetal mortality rates in DKA were reported as high as 30-60%, but advances in maternal critical care and obstetric management have reduced these rates to approximately 9-35% in contemporary series. Maternal mortality is now rare, though still possible in cases of profound acidosis or delayed recognition [3].

Pregnancy induces profound metabolic changes that predispose individuals with diabetes to rapid ketone production. Placental hormones, including human placental lactogen, progesterone, cortisol, and placental growth hormone, progressively increase insulin resistance during the second and third trimesters of pregnancy [4]. This physiologic insulin resistance promotes lipolysis and mobilization of free fatty acids, which are subsequently converted by the liver into ketone bodies such as acetoacetate and beta-hydroxybutyrate. As pregnancy advances, maternal metabolism increasingly relies on fatty acid oxidation to preserve glucose availability for the developing fetus, thereby facilitating ketosis when insulin availability becomes insufficient [5].

Additional physiologic mechanisms further increase susceptibility to metabolic acidosis during pregnancy. Progesterone-mediated stimulation of maternal ventilation produces a chronic respiratory alkalosis that lowers circulating bicarbonate concentrations. This reduction in maternal buffering capacity limits the ability to neutralize accumulating organic acids, allowing relatively modest increases in ketone production to result in clinically significant acidemia [6]. At the same time, continuous transplacental glucose transport to the fetus may maintain maternal serum glucose levels within the normal range despite ongoing hepatic gluconeogenesis and ketogenesis, contributing to the characteristic euglycemic biochemical profile observed in EDKA [7].

Clinical recognition of EDKA may therefore be challenging. Symptoms such as dyspnea, nausea, fatigue, or tachycardia are frequently nonspecific and may overlap with normal physiologic changes of pregnancy or with obstetric complications such as preeclampsia [8]. Furthermore, several precipitating factors, including infection, dehydration, prolonged fasting, vomiting, corticosteroid administration, insulin omission, and the metabolic stress associated with labor or surgical intervention, have been identified as triggers for ketoacidosis in pregnancy [5,9]. Because fetal acid-base status closely reflects maternal metabolic conditions, untreated ketoacidosis can rapidly impair uteroplacental perfusion and fetal oxygen delivery, increasing the risk of fetal distress, preterm delivery, and intrauterine demise [3].

In high-acuity obstetric settings, hemodynamic instability, emergent operative interventions, and anesthetic considerations may dominate clinical attention, making subtle metabolic abnormalities more difficult to identify. This case illustrates how overlapping obstetric and metabolic stressors can mask EDKA and contribute to abrupt intraoperative cardiovascular collapse.

## Case presentation

Admission and initial evaluation

An 18-year-old gravida 1, para 0 woman at 35 weeks and 4 days of gestation was transferred to our institution for management of preeclampsia with severe features, evidenced by severe hypertension and signs of pulmonary edema. Her medical history was significant for type 1 diabetes mellitus, treated with insulin aspart and insulin glargine. The patient reported poor adherence to insulin therapy for more than seven days prior to presentation.

On admission, she complained of dyspnea and progressive peripheral edema. Vital signs revealed hypertension and mild tachycardia. Physical examination was notable for bilateral lower-extremity edema without focal neurologic deficits. Initial laboratory evaluation revealed metabolic acidosis with compensatory hypocapnia and ketonuria, suggesting early metabolic derangement despite near-normal serum glucose levels (Table 1). 

**Table 1:** Laboratory results at admission pCO₂: partial pressure of carbon dioxide, ALT: alanine aminotransferase, AST: aspartate aminotransferase.

Parameter	Reference Range	At Admission	Interpretation
pH, arterial	7.35-7.45	7.32 (L)	Mild acidosis
pCO₂, arterial	35-45 mmHg	30 (L)	Compensatory hypocapnia
Bicarbonate	23-31 mmol/L	17 (L)	Metabolic acidosis
Base excess	−2 to +2 mmol/L	2	Within normal limits
Sodium	135-145 mmol/L	134 (L)	Mild hyponatremia
Potassium	3.5-5.1 mmol/L	3.4 (L)	Hypokalemia
Chloride	98-107 mmol/L	102	Normal
Blood urea nitrogen	7-20 mg/dL	9	Normal
Creatinine	0.6-1.2 mg/dL	0.7	Normal renal function
Ketones, urine	Negative	Trace	ketonuria
Glucose	70-120 mg/dL	156	Hyperglycemia
Hemoglobin	12.0-15.3 g/dL	9.3 (L)	Anemia
Platelets	140-400 k/µL	213	Normal
ALT	10-35 U/L	10	Normal
AST	10-35 U/L	7	Normal
Bilirubin, total	≤1.2 mg/dL	<0.2 mg/dL	Normal

These findings suggested early metabolic instability in the setting of insulin nonadherence, while preserved renal function indicated a primarily metabolic rather than renal etiology. Fetal ultrasound demonstrated a cephalic fetus with a reassuring fetal heart rate and polyhydramnios. The patient was started on intravenous magnesium sulfate for seizure prophylaxis (4 g loading dose followed by continuous infusion at 2 g/hour) and subcutaneous sliding-scale insulin therapy for glycemic control. After admission, glucose levels remained within the normal range. An echocardiogram was performed on the day of surgery because pulmonary edema was suspected. The results were normal, with no evidence of cardiac dysfunction and no indication for modification of the treatment plan.

Preoperative course

An epidural catheter was placed for labor analgesia without complications. However, during the latent phase of labor, recurrent late fetal heart rate decelerations were observed on continuous fetal monitoring. Due to concern for nonreassuring fetal status, the decision was made to proceed with emergent cesarean delivery.

The patient was re-evaluated by the anesthesia team in her room, where she was noted to be tachypneic and seated at 90 degrees, with the remaining vital signs within normal limits at that time. The epidural catheter was functioning at a rate of 8 mL/hour, with sensory levels at T10. The patient reported vomiting during the day and was therefore considered to have a full stomach. She was moved emergently to the operating room after administration of a 10 mL bolus of 1% lidocaine with 1 mL of 8.4% bicarbonate through the epidural catheter, and the epidural infusion was discontinued.

Preoperative laboratory testing revealed electrolyte abnormalities, including hypokalemia and hypermagnesemia, along with mild hyperglycemia and an elevated hemoglobin A1c, indicating poor chronic glycemic control (Table 2). Owing to the urgency of the diagnosis, additional laboratory tests were not obtained, and the patient was moved urgently to the operating room.

**Table 2:** Preoperative laboratory results

Parameter	Reference Range (Units)	Preoperative	Interpretation
Potassium	3.5-5.1 mmol/L	3.4	Hypokalemia
CO₂	22-32 mmol/L	24	Normal
Anion Gap	7-15 mmol/L	15	Upper normal
Magnesium	1.5-2.6 mg/dL	6 (H)	Hypermagnesemia
Glucose (POCT)	70-120 mg/dL	129 (H)	Hyperglycemia
Hemoglobin A1c	4.0-5.6 %	7.8 (H)	Chronic hyperglycemia

These findings reflected a metabolically vulnerable physiologic state prior to surgery. The remaining laboratory parameters not shown in the table, including platelet count and hepatic enzymes, were within normal limits.

Intraoperative course

The patient was positioned on the operating table, and an emergent cesarean section was initiated following the surgical timeout. The adequacy of the epidural anesthesia was assessed with a skin clamp, which elicited no painful response, and the incision was made. After entry into the abdominal cavity, however, the patient began to report sharp pain that did not improve despite additional epidural boluses. The decision was therefore made to convert to general endotracheal anesthesia using rapid-sequence induction. Propofol 200 mg and succinylcholine 100 mg were administered as intravenous boluses, resulting in atraumatic intubation with immediate confirmation of bilateral ventilation.

Within three minutes of induction, the newborn was delivered with Apgar scores of 8 and 9 at 1 and 5 minutes, respectively, and was admitted to the NICU for observation.

Following delivery of the newborn and umbilical cord clamping, an oxytocin infusion was initiated at 900 mL/hour (54 units/hour). Despite the high-dose intravenous oxytocin, the uterus remained boggy, and the patient developed active, uncontrolled uterine bleeding. The surgical team requested tranexamic acid (1 g intravenously), which was administered while uterine traction was applied. No additional uterotonic agents were given at that time.

Shortly thereafter, the anesthesia team noted that the patient had developed acute bradycardia, with heart rates decreasing to approximately 50 beats per minute, while blood pressure initially remained stable. An initial dose of glycopyrrolate (0.2 mg IV) produced no improvement. A second dose of glycopyrrolate (0.2 mg IV) was administered, again without change in heart rate, although blood pressure remained within normal limits.

Magnesium-related cardiac conduction effects were suspected; therefore, the magnesium sulfate infusion was discontinued, and 1 g of intravenous calcium gluconate was administered. In spite of these interventions, the patient developed persistent bradycardia and progressive hypotension, prompting administration of atropine (0.5 mg intravenously).

Cardiac arrest and resuscitation

Despite the pharmacologic interventions already administered, the attending anesthesiologist was unable to palpate a carotid pulse and immediately alerted the surgical and anesthesia teams. A Code Lavender was activated, and the patient was found to be in pulseless electrical activity (PEA). High‑flow 100% oxygen was delivered via the anesthesia machine, and chest compressions were continued with effective, high‑quality technique throughout the resuscitation effort. Cardiopulmonary resuscitation (CPR) was initiated without delay, and 1 mg of intravenous epinephrine was administered. Within approximately one minute, return of spontaneous circulation (ROSC) was achieved, evidenced by the reappearance of palpable pulses, a rapid rise in blood pressure, and a marked increase in end‑tidal carbon dioxide. Following stabilization, an arterial line was placed to facilitate continuous hemodynamic monitoring, and the surgical team proceeded to secure hemostasis.

After the patient was stabilized, the anesthesia and surgical teams systematically evaluated potential causes of the cardiac arrest and explored the differential diagnoses. Arterial blood samples were obtained and analyzed at the bedside using an i‑STAT device in the operating room. Post‑arrest laboratory assessment revealed profound metabolic derangement, including severe high‑anion‑gap metabolic acidosis, critically low bicarbonate levels, and worsening electrolyte abnormalities (Table 3). A bolus of 50 mEq of sodium bicarbonate was administered in response to the marked acidosis. An estimated blood loss of approximately 1 liter was calculated, and appropriate fluid resuscitation was provided to maintain hemodynamic stability. With hemostasis achieved and uterine bleeding controlled, the decision was made to transfer the patient to the ICU for ongoing resuscitation and metabolic stabilization. The remainder of the procedure proceeded uneventfully, and the patient remained hemodynamically stable without the need for vasopressor support.

**Table 3:** Laboratory results post cardiac arrest pCO₂: partial pressure of carbon dioxide, POCT: point-of-care testing, ALT: alanine aminotransferase, AST: aspartate aminotransferase.

Parameter	Reference Range (Units)	After Cardiac Arrest	Interpretation
pH, arterial	7.350-7.450	6.989 (LL)	Extremely severe acidosis
pCO₂, arterial	35.0-45.0 mmHg	35.8	Normal
Base excess, arterial	-2.0 to 2.0 mmol/L	-21.8 (L)	Severe metabolic acidosis
Bicarbonate, whole blood	23.0-31.0 mmol/L	8.2 (L)	Markedly low; metabolic acidosis
Ketones, urine	Negative	40	Ketonuria
Potassium	3.5-5.1 mmol/L	3.2 (L), 3.3 (L)	Hypokalemia
CO₂	22-32 mmol/L	8 (L)	Severe hypocapnia
Anion gap	7-15 mmol/L	23 (H), 18 (H)	High anion gap metabolic acidosis
Magnesium	1.5-2.6 mg/dL	8.1 (HH)	Severe hypermagnesemia
Glucose (POCT)	70-120 mg/dL	123 (H)	Hyperglycemia
Hemoglobin	12.0-15.3 g/dL	8.9 (L)	Anemia
Platelets	140-400 k/µL	284	Normal
ALT	10-35 U/L	30	Normal
AST	10-35 U/L	9	Low
Bilirubin, total	≤1.2 mg/dL	0.2	Normal

Postoperative ICU course

Sodium bicarbonate therapy, insulin infusion, and potassium replacement were initiated. The patient was transferred to the intensive care unit, intubated, sedated, and hemodynamically stable. Repeat laboratory evaluation demonstrated ketoacidosis despite normal glucose levels. 

The patient remained intubated for 48 hours until compensation of the acid-base imbalance and normalization of glucose levels were achieved. She recovered without sequelae. The patient did not require vasopressors during her ICU admission. Her DKA was managed with bicarbonate and an insulin infusion based on a sliding scale.

Written informed consent was obtained from the patient for publication of this case report and the accompanying clinical details. All identifying information has been removed or anonymized to protect patient confidentiality, and the report was prepared in accordance with institutional and international ethical standards for human subjects.

## Discussion

DKA during pregnancy is a high-risk metabolic emergency, and euglycemic presentations are increasingly recognized. Physiologic adaptations of late gestation, including increased insulin resistance, enhanced lipolysis, and reduced buffering capacity, predispose pregnant patients with type 1 diabetes to ketosis even without marked hyperglycemia, driven in part by increased caloric demand and augmented glucose uptake by the fetus and placenta [10-13]. Up to one-third of DKA cases in pregnancy may be euglycemic, and this atypical biochemical profile can delay recognition, particularly in high-acuity obstetric settings where other complications dominate immediate clinical attention [2,4].

This patient demonstrated several well-described risk factors for EDKA: advanced gestation, prolonged interruption of insulin therapy, and metabolic acidosis despite normoglycemia. Additional contributors included vomiting and reduced caloric intake, both of which have a bidirectional relationship with ketoacidosis, serving as both triggers and manifestations of worsening ketosis. Her admission glucose level of 117 mg/dL did not initially suggest severe metabolic decompensation, illustrating how euglycemia may obscure the diagnosis of DKA [5-8]. Although trace ketonuria was present at admission, the absence of overt acidosis likely contributed to delayed recognition. Poor glycemic control was also not evident from point-of-care glucose checks alone, consistent with reports showing that EDKA can occur even in patients using insulin pumps, notwithstanding technological advances intended to reduce the risk of ketoacidosis. The resulting acidosis posed a threat to fetal well-being, as reflected by persistent decelerations that prompted emergent cesarean delivery, although the newborn ultimately had reassuring Apgar scores.

From an anesthetic perspective, the primary goal in patients with suspected or evolving EDKA is early identification of acidosis and ketosis to guide perioperative management. Severe preeclampsia likely compounded physiologic vulnerability by contributing to endothelial dysfunction, altered intravascular volume status, and reduced cardiovascular reserve [6-9]. Symptom overlap between preeclampsia and metabolic decompensation, such as dyspnea, malaise, and edema, may further obscure early recognition. In previously reported cases, the coexistence of ketoacidosis and preeclampsia has increased the likelihood of requiring general anesthesia due to pulmonary edema or hemodynamic instability [10]. Although ketoacidosis does not inherently contraindicate regional anesthesia, successful use of combined spinal-epidural techniques has been described even in uncompensated DKA [14-18]. However, diabetes and acidosis increase the risk of aspiration during general anesthesia, adding complexity to airway management.

Given the abrupt intraoperative cardiovascular collapse, a broad differential diagnosis was appropriately considered. Amniotic fluid embolism (AFE) was evaluated due to its classic presentation of sudden cardiovascular collapse, hypoxemia, and coagulopathy. In this case, the absence of severe hypoxemia, disseminated intravascular coagulation, or persistent hemodynamic instability made AFE unlikely [7]. Magnesium sulfate toxicity was also considered, particularly given the ongoing infusion. Although serum magnesium levels were elevated, they remained below concentrations typically associated with respiratory depression or significant cardiac conduction abnormalities [8].

Obstetric hemorrhage was another potential cause of hemodynamic instability. However, estimated blood loss and rapid surgical control did not support severe hypovolemia as the primary etiology. In the context of ongoing ketoacidosis, even moderate bleeding may exacerbate hemodynamic compromise due to relative hypovolemia and vasodilation. Reactions to tranexamic acid (TXA), including hypotension or hypersensitivity, have been described, although they are uncommon with standard obstetric dosing. No features of anaphylaxis or refractory hypotension were present [4].

Anesthetic-related complications were also explored. Excessive opioid or volatile anesthetic exposure can cause cardiovascular depression, but the anesthetic course did not support this mechanism. Pregnancy-associated cardiomyopathy was considered, particularly given the abrupt collapse; however, the absence of prior cardiac disease, rapid return of spontaneous circulation, and normal postoperative echocardiogram made this diagnosis unlikely. Hyperchloremic metabolic acidosis from large-volume sodium chloride infusion was another consideration, but this condition typically presents with a normal anion gap. In this case, the presence of ketonuria and a markedly elevated anion gap was inconsistent with hyperchloremic acidosis [5].

Ultimately, laboratory evaluation demonstrated severe metabolic acidosis with ketosis despite normoglycemia, supporting the diagnosis of EDKA. The coexistence of multiple physiologic stressors, including preeclampsia, surgical stress, magnesium exposure, and acute obstetric intervention, likely contributed to the severity and complexity of the presentation. This case highlights how EDKA may be overlooked when glucose levels fall within the normal range, particularly in the presence of competing obstetric and anesthetic concerns.

Early evaluation of acid-base status and ketone levels is essential in pregnant patients with diabetes, especially when insulin omission or physiologic stressors are present. Intraoperative cardiovascular collapse requires rapid assessment of metabolic causes, as delayed recognition may worsen maternal and fetal outcomes. Multidisciplinary coordination between obstetrics, anesthesia, endocrinology, and critical care teams is crucial to optimize management.

Key clinical messages

EDKA may occur in pregnancy despite normal glucose levels and can be easily overlooked. Insulin omission and late gestation significantly increase susceptibility to ketosis. Intraoperative cardiovascular collapse requires rapid evaluation of metabolic causes. Early recognition and multidisciplinary management are essential to optimize outcomes.

Limitations

Preoperative ketone levels were not available, limiting assessment of the onset and progression of ketosis. Lactate and beta-hydroxybutyrate quantification were not obtained, which may have further clarified the metabolic profile. Multiple overlapping clinical factors, including preeclampsia, anesthesia, uterine manipulation, and magnesium exposure, prevent definitive attribution of the cardiac arrest to a single cause.

## Conclusions

Euglycemic diabetic ketoacidosis in pregnancy is an uncommon but serious condition that may present without hyperglycemia, delaying recognition. In this patient, insulin omission, advanced gestation, and preeclampsia created a high-risk metabolic environment that contributed to severe acidosis and intraoperative cardiovascular collapse. Early evaluation of acid-base status, prompt correction of metabolic abnormalities, and coordinated multidisciplinary care are essential to improving maternal outcomes in high-risk obstetric patients.
